# Feeding Healthy Beagles Medium-Chain Triglycerides, Fish Oil, and Carnitine Offsets Age-Related Changes in Serum Fatty Acids and Carnitine Metabolites

**DOI:** 10.1371/journal.pone.0049510

**Published:** 2012-11-07

**Authors:** Jean A. Hall, Dennis E. Jewell

**Affiliations:** 1 Department of Biomedical Sciences, College of Veterinary Medicine, Oregon State University, Corvallis, Oregon, United States of America; 2 Pet Nutrition Center, Hill’s Pet Nutrition, Inc, Topeka, Kansas, United States of America; Auburn University, United States of America

## Abstract

The purpose of this study was to determine if feeding dogs medium-chain triglycerides (MCT), fish oil, and L-carnitine enriched foods offsets age-associated changes in serum fatty acids (FA) and carnitine metabolites. Forty-one healthy Beagles, mean age 9.9 years (range 3.1 to 14.8), were fed control or one of two treatment foods for 6 months. All foods were complete and balanced and met the nutrient requirements for adult dogs, and had similar concentrations of moisture, protein, and fat (approx. 7.4%, 14.0%, and 18.1%, respectively). The treatment diets both contained added L-carnitine (300 mg/kg) and 0.6% (treatment food 1) or 1.5% (treatment food 2) added fish oil. Treatment food 2 also had increased MCT from coconut oil, added corn oil, and reduced animal fat. Composition of serum FA was determined by gas chromatography of FA methyl esters. Metabolomic profiles of serum samples were determined from extracted supernatants that were split and run on GC/MS and LC/MS/MS platforms, for identification and relative quantification of small metabolites. Body composition was determined by dual energy x-ray absorptiometry. Among dog groups, there was no change in total-lean-body weight, or in serum total protein and serum albumin concentrations, based on time or dietary treatment. Serum concentrations of carnitine metabolites were decreased in geriatric (>7 years) vs. mature adult (≤7 years) dogs, and supplementation with L-carnitine attenuated the effects of aging. The ratio of PUFA to SFA was significantly greater in mature dogs at baseline (*P*≤*0.05)*. Serum concentrations of eicosapentaenoic and docosahexaenoic FA increased in a dose-dependent manner. Dogs consuming treatment food 2 also had increased serum concentrations of lauric and myristic FA, and decreased concentrations of SFA, MUFA, and arachidonate (all *P*≤0.05) and their PUFA to SFA ratio increased. In summary, dietary MCT, fish oil, and L-carnitine counterbalanced the effects of aging on circulating concentrations of these compounds.

## Introduction

Nutritional intervention with dietary supplements offers a means of altering the age-related decline in energy metabolism that is recognized in older animals (reviewed in [1]). Carnitine is a necessary nutrient in energy production and its content decreases with aging [2], [3]. Carnitine performs an obligate role in mitochondrial oxidation of long-chain fatty acids (FA) through the action of specialized acyltransferases [4]. Briefly, L-carnitine is needed to transport long-chain FA from the cytosol to sites of beta-oxidation in the mitochondria. Acetate is the by-product of beta-oxidation and provides usable energy via the citric acid cycle. Under normal conditions, the availability of carnitine is not a limiting step in beta-oxidation, although an increase in carnitine content may increase the rate of FA oxidation, permitting a reduction of glucose utilization, preserving muscle glycogen content, and ensuring maximal rates of oxidative ATP production [5]. Older dogs with age-related progressive renal dysfunction may be polyuric and uremic. Loss of carnitine through dialytic membranes has been documented in hemodialysis patients with end-stage kidney disease, resulting in potential carnitine depletion [6]. Carnitine supplementation has been shown to counteract such alterations and may be associated with improvements in metabolic and clinical complications seen in uremic patients (reviewed in [6], [7]).

Serum levels of specific FA are negatively correlated with age in humans [8]. In a large (n = 1123) community-based study of persons aged 20 to 98 years, mean plasma concentration of total FA tended to be higher at older ages, yet the percentage of (n-3) and (n-6) FA were significantly lower in older participants, which may explain the mild proinflammatory state that is often found in the elderly [8].

Fat is an important dietary component, serving both as a source of energy and as a supplier of essential FA. We have shown in previous studies that feeding healthy dogs with different concentrations of dietary fish oil alters circulating concentrations of FA [9]–[13]. In general, the long-chain (n-3) polyunsaturated fatty acids (PUFA) from fish oil are considered anti-inflammatory and immunomodulatory [14]. At sufficient levels of incorporation, eicosapentaenoic acid [EPA, 20∶5 (n-3)] and docosahexaenoic acid [DHA, 22∶6 (n-3)] influence the physical nature of cell membranes and membrane protein-mediated responses, lipid-mediator generation, cell signaling, and gene expression in many different cell types [15]. The PUFA from fish oil, and in particular eicosanoids derived from EPA, may protect against excessive inflammatory reactions, which may be exacerbated by eicosanoids derived from the (n-6) PUFA arachidonic acid [AA, 20∶4 (n-6)]. There is some, albeit limited, data to support the notion that (n-3) PUFA ameliorate clinical symptoms in human patients with diseases characterized by active inflammation [16]. In dogs, there is evidence to suggest that (n-3) FA have beneficial effects in the treatment of osteoarthritis [17].

The products of lipid digestion undergo an interesting journey before they reach the bloodstream. First, lipolytic by-products are solubilized in micelles within the intestinal lumen. They then diffuse from the micelle across the enterocyte brush border membrane and enter the cytoplasm of the cell. Within enterocytes, triglycerides are resynthesized. The resynthesized triglycerides and phospholipids, together with cholesterol are combined with apoprotein to form chylomicrons. Chylomicrons leave the cell by exocytosis and enter the lymph via the lacteals in the villi. Finally, they reach the bloodstream via the thoracic duct.

Medium-chain triglycerides (MCT) contain intermediate chain length FA, which are water soluble and can be absorbed directly without depending on micelle formation. They also pass through enterocytes without being resynthesized into triglycerides. They do not take part in chylomicron formation and are absorbed directly into the portal blood. The MCT also do not rely on carnitine for transport across the inner mitochondrial membrane. The names of the medium-chain FA (and the corresponding number of carbons) found in MCT are caproic acid [C6∶0], caprylic acid [C8∶0], capric acid [C10∶0] and lauric acid [C12∶0]. Coconut oil is composed of approximately 66% MCT.

Previous human studies have shown that inclusion of 10% fish oil in mixed medium- and long-chain triacylglycerol emulsions increases plasma triacylglycerol clearance and induces rapid EPA incorporation into blood-cell phospholipids [18]. The metabolism of lipid emulsions is similar to that of chylomicrons with intravascular lipolysis by lipoprotein lipase followed by tissue uptake of remnant particles [19]. The fast hydrolysis of MCT releases large amounts of medium-chain FA, which are rapidly oxidized by many tissues sparing (n-3) FA from entering oxidative pathways. Rapid lipolysis of MCT also leads to the formation of small-sized remnant particles enriched with (n-3) FA that can be readily taken up by endocytosis [20], allowing increased EPA and DHA FA incorporation into membrane phospholipids [21].

The purpose of this study was to determine if dogs consuming a complete and balanced adult-food base formula with added L-carnitine, MCT, and (n-3) FA from fish oil are able to maintain total-lean-body weight, total serum protein and albumin concentrations, and achieve desired alterations in serum FA profiles and carnitine metabolite concentrations. We hypothesized that serum carnitine and long chain (n-3) FA concentrations decrease with age, and that these changes could be offset by nutritional intervention. We were able to document age-related changes in the PUFA to SFA ratio and in carnitine metabolite concentrations that could be counterbalanced by dietary enrichment with these compounds.

## Materials and Methods

### Dogs and Ethics Statement

The study protocol was reviewed and approved by the Institutional Animal Care and Use Committee, Hill’s Pet Nutrition, Inc., Topeka, KS, USA (Permit Number: 09-487). All dogs were immunized against canine distemper, adenovirus, parvovirus, bordetella, and rabies, and none had chronic systemic disease on the basis of results of physical examination, complete blood count determination, serum biochemical analyses, urinalysis, and fecal examination for parasites. Dogs were housed in pairs in indoor runs or in spacious rooms with natural light that varied with seasonal changes. All dogs were exercised daily, and were provided with regular opportunities for socialization and environmental enrichment. Dogs experienced behavioral enrichment through interactions with each other, by daily interaction and play time with caretakers, and by daily opportunities to run and exercise outside with access to toys.

Forty one healthy Beagle dogs with mean age of 9.9 years (range 3.1 to 14.8 years) were included in this study. Equal numbers of females (n = 21; ovariohysterectomized) and males (n = 20; neutered) were randomized to 3 study groups. Initial body weight, mean ± SEM, was 12.4±0.4 kg. Exclusion criteria included the inability to eat dry food and/or any diagnosed disease condition. The criterion for removal from the study was development of any condition whereby removal would benefit the animal. Three animals were removed during the study for unrelated reasons: bladder carcinoma, surgery for intervertebral disc disease, and foot abscess with fever. All other animals remained healthy and completed the study.

To evaluate the influence of age, age categories were defined as 7 years and under (mature adults), or older than 7 years (geriatric adults). This resulted in reasonably balanced groups at the end of the study with 18 animals in the mature adult group (control food n = 5; food with increased (n-3) FA content n = 8; food with increased (n-3) and (n-6) FA food that was enriched in MCT n = 5) and 20 animals in the geriatric adult group (control food n = 8; food with increased (n-3) FA content n = 4; food with increased (n-3) and (n-6) FA food that was enriched in MCT n = 8).

### Foods

Prior to beginning the study, all dogs were consuming the same non-test food for 30 days (**Table 1**). This pre-trial food was a complete and balanced adult canine food [22], although relatively low in fat.

**Table 1:** Food composition of pre-trial food, control food*, and two dietary treatment foods.†

	Pre-trial food	Control Food	Increased (n-3)FA Food	Increased (n-3) and(n-6) FA Food
Added Fish Oil, %	0	0	0.6	1.5
Added L-carnitine, mg/kg	0	0	300	300
Added Coconut andCorn Oils, (+,–)	–	–	–	+
Moisture	6.38	6.88	7.86	7.58
Protein	18.25	13.66	14.12	14.09
Fat	8.07	18.15	17.89	18.32
Atwater Energy§ (kcal/kg)	3,484	3,960	3,921	3,959
Ash	4.20	3.90	4.38	3.97
Crude Fiber	1.4	2.0	1.3	1.5
Calcium	0.89	0.70	0.75	0.72
Phosphorus	0.73	0.28	0.31	0.29
Sodium	0.12	0.2	0.2	0.2
Capric acid [10∶0]	0.00	0.00	0.01	0.12
Lauric acid [12∶0]	0.00	0.01	0.01	0.87
Myristic acid [14∶0]	0.05	0.17	0.18	0.46
Palmitic acid [16∶0]	1.38	3.60	3.45	2.26
Stearic acid [18∶0]	0.49	1.67	1.53	0.76
Arachidic acid [20∶0]	0.02	0.03	0.03	0.04
LA [18∶2 (n-6)]	2.10	3.07	3.09	4.61
αLA [18∶3 (n-3)]	0.08	1.27	1.35	1.63
AA [20∶4 (n-6)]	0.03	0.08	0.08	0.06
EPA [20∶5 (n-3)]	0.00	0.01	0.10	0.23
DHA [22∶6 (n-3)]	0.00	0.00	0.07	0.16
∑ SFA£	1.97	5.56	5.40	4.70
∑ MUFA¥	2.44	6.81	6.21	5.26
∑ PUFA¶	2.26	4.52	4.65	6.65
∑ (n-6) FA#	2.17	3.22	3.15	4.59
∑ (n-3) FA‡	0.09	1.30	1.49	2.06
(n-6):(n-3) ratio	24.11	2.48	2.11	2.23

* Prescription Diet® k/d®, Hill’s Pet Nutrition, Inc.

† All analytical values are expressed as percentage of food as fed, unless otherwise indicated.

§ Calculated from analyticals using modified Atwater numbers (kcal/g of 3.5 for protein, 8.5 for fat and 3.5 for nitrogen free extract).

£ Sum of the saturated fatty acids: 8∶0+10∶0+11∶0+12∶0+14∶0+15∶0+16∶0+17∶0+18∶0+20∶0+22∶0+24∶0.

¥ Sum of the monounsaturated fatty acids: 14∶1+15∶1+16∶1+17∶1+18∶1+20∶1+22∶1+24∶1.

¶ Sum of the polyunsaturated fatty acids: 18∶2(n-6)+18∶3(n-6)+18∶3(n-3)+18∶4(n-3)+20∶2(n-6)+20∶3(n-6)+20∶3(n-3)+20∶4(n-6)+20∶4(n-3)+20∶5(n-3)+21∶5(n-3)+22∶2(n-6)+22∶4(n-6)+22∶5(n-6)+22∶5(n-3)+22∶6(n-3).

# Sum of the (n-6) fatty acids.

‡ Sum of the (n-3) fatty acids.

Three test foods were prepared by Hill’s Pet Nutrition, Inc.: a control food (Hill’s® Prescription Diet® k/d®, Hill’s Pet Nutrition, Inc.) and two foods with added L-carnitine but different concentrations of FA. Both experimental foods were Prescription Diet® k/d® base food with added L-carnitine (300 mg/kg as fed) and fish oil (0.6 or 1.5% as fed). The test food with 1.5% fish oil and added MCT from coconut oil also had reduced AA because of a reduction in animal fat in the formulation. The reduced animal fat was replaced with plant oils (2% coconut oil and 7% corn oil). Food composition of the three diets is summarized in **Table 1**.

All test foods contained similar concentrations (within analytical variance of targets) of protein, fat, calcium, phosphorus, and sodium, and were isocaloric. The control food lacked appreciable quantities of long chain EPA and DHA, and medium-chain capric [10∶0], lauric [12∶0], and myristic [14∶0] FA. Control food was richest in palmitic [16∶0] and stearic [18∶0] FA. Treatment food 1 was richer in EPA and DHA than control food, but comparable in medium-chain FA content. Treatment food 2 contained increased concentrations of EPA and DHA, as well as increased concentrations of medium-chain FA, and linoleic [LA, 18∶2 (n-6)] and α-linolenic [αLA, 18∶3 (n-3)] FA. Treatment food 2 also contained the least amounts of palmitic, stearic, and AA FA. All foods had relatively the same (n-6) to (n-3) FA ratio, which was approximately 2.3∶1.

### Study Design and Measurements

The study design was a three treatment study, whereby dogs were fed either control food or one of two treatment foods. Study duration was 6 months. All dogs had access to electronic feeders where fresh food was offered daily with amounts calculated to maintain body weight; water was available ad libitum. Daily food intake (g/d) was recorded for each dog.

For each dog, data were collected initially and again after consuming foods for one, three, and six months. Blood was collected to measure serum biochemistries, serum FA concentrations, and serum metabolomic profiles.

Fatty acid composition of the experimental foods was determined by a commercial laboratory (Eurofins Scientific, Inc., Des Moines, IA) by gas chromatography of FA methyl esters. Fatty acid concentrations are expressed as g/100 g of FAs as fed. The sum of dietary saturated fatty acids (SFA) was determined as follows: 8∶0+10∶0+11∶0+12∶0+14∶0+15∶0+16∶0+17∶0+18∶0+20∶0+22∶0+24∶0. The sum of dietary monounsaturated fatty acids (MUFA) was determined as follows: 14∶1+15∶1+16∶1+17∶1+18∶1+20∶1+22∶1+24∶1. The sum of dietary PUFA was determined as follows: 18∶2(n-6)+18∶3(n-6)+18∶3(n-3)+18∶4(n-3)+20∶2(n-6)+20∶3(n-6)+20∶3(n-3)+20∶4(n-6)+20∶4(n-3)+20∶5(n-3)+21∶5(n-3)+22∶2(n-6)+22∶4(n-6)+22∶5(n-6)+22∶5(n-3)+22∶6(n-3).

Fatty acid composition of serum samples was also determined by gas chromatography of FA methyl esters, with minor modifications [17] of the Folch et al. [23] method. Fatty acid concentrations in serum, determined using this methodology, were expressed as mg/dL.

Metabolomic profiles of serum samples were determined by a commercial laboratory (Metabolon; Durham, NC) using a proprietary solvent extraction method. Extracted supernatant was split and run on gas chromatography and liquid chromatography mass spectrometer platforms [24] in a randomized order. Gas chromatography (for hydrophobic molecules) and liquid chromatography (for hydrophilic molecules) were used to identify and provide relative quantification of small metabolites present in serum samples. Endogenous biochemicals included amino acids, peptides, carbohydrates, lipids, (e.g., FA and lysophospholipids), nucleotides, cofactors and vitamins, and xenobiotics. Data for each individual compound were normalized by calculating the median values for each run-day block (block normalization). This minimized any inter-day instrument gain drift, but did not interfere with intra-day sample variability. Missing values were assumed to be below the level of detection for that compound with the instrumentation used. Missing values (if any) were imputed with the observed minimum for that particular compound. Imputed values were added after block-normalization.

Changes in body mass and composition were assessed by dual-energy x-ray absorptiometry (DXA-QDR-4500, Hologic, Inc., Waltham, MA) scan analysis initially and after six months of food consumption. Data were analyzed by the software supplied by the manufacturer. Total-, fat-, and lean-body mass were determined.

### Statistical Analyses

The analyses for serum biomarkers and changes in body mass and composition were performed using a repeated measures regression model in Statistical Analysis Software version 9.2 (SAS Institute, Cary, NC) for initial, final, and change between final and initial concentrations. Mean separation was performed using animal age, food formula and time on treatment as independent variables. Significance was accepted as *P*≤0.05, unless otherwise specified.

For metabolomic data, serum values from the end of the pretrial feeding period (day 0) were used such that each animal served as its own control, and data are presented relative to day 0 as fold change. All values were log-transformed prior to statistical analysis. Welch’s two-way ANOVA and two-sample t-test analyses were applied to log-transformed data. A *q*-value cutoff of 0.10 was used. This value provides an estimate of the proportion of false discoveries within each of the individual contrasts and based on this, 10% of the compounds determined to be significant within each of the individual contrasts may possibly represent false positives. Main effects of animal age, diet, time, and diet by time interaction, as well as *t*-test comparisons were considered significant if *P*<0.05 and *q*<0.10. To investigate the relationship between creatinine and carnitine moities, correlation coefficients were measured between these response variables.

## Results

### The Effect of Dietary Treatment on Total-, Fat-, and Lean-body Weights

There were no differences in average daily food intake among dogs of the 3 dietary treatment groups. All dogs gained weight after consuming the dietary treatment foods for 6 months (*P*<0.01), but there were no differences in total-body weight gain based on dietary treatment (**Table 2**). Total-fat-body weight increased in dogs from all 3 treatment groups after 6 months of food consumption. There were no changes in total-lean-body weight after consuming the dietary treatment foods for 6 months based on time or dietary treatment.

**Table 2:** Body weight and composition indices, and selected serum biochemistries from serum biochemical profiles, of dogs at baseline (initial) and after consuming control* or treatment foods for 194 days (mean ± SEM).

	Control Food	Increased (n-3)FA Food	Increased(n-3) and(n-6) FA Food	Two-way ANOVA Analysis(*P* values)		
**Number of Animals, N**	13	12	13	**Diet** **Main Effect**	**Time Main Effect**	**Diet by Time Main Effect**
**Added Fish Oil, %**	0	0.6	1.5			
**Added L-carnitine, mg/kg**	0	300	300			
**Added Coconut and Corn Oils, Reduced Animal Fat (+,–)**	–	–	+			
**Body weight and composition indices:**						
Total-body weight† (kg)InitialFinal	11.83±0.8312.65±0.87	12.53±0.5113.55±0.54	13.00±0.5513.77±0.57		<0.01	
Total-fat-body weight† (g)InitialFinal	3406±1654506±281	3781±1694900±185	4426±3165108±286		<0.01	
Total-lean-body weight† (g)InitialFinal	8032±6887843±651	8346±5168301±533	8271±4378321±446			
**Serum biochemistries:**						
Albumin (mg/dL)InitialFinal	3.46±0.083.53±0.07	3.53±0.073.57±0.06	3.49±0.063.62±0.05			
Total Protein (mg/dL)InitialFinal	5.65±0.095.70±0.08	5.73±0.115.78±0.07	5.71±0.065.86±0.07			
Urea Nitrogen (mg/dL)InitialFinal	9.88±0.687.96±0.50	10.23±0.657.73±0.54	10.61±0.588.25±0.41		<0.01	
Creatinine (mg/dL)InitialFinal	0.66±0.050.63±0.04	0.61±0.050.68±0.06	0.66±0.040.79±0.05			
Phosphorous (mg/dL)InitialFinal	3.48±0.204.36±0.19	3.88±0.224.31±0.25	3.66±0.224.09±0.29		0.03	
Triglycerides (mg/dL)InitialFinal	100±33195±18	88±14236±30	73±15184±13		<0.01	
Cholesterol (mg/dL)InitialFinal	187±9235±9	205±10250±20	201±11217±7		<0.01	

* PrescriptionDiet® k/d®, Hill’s Pet Nutrition, Inc.

† Body mass and composition were determined by dual-energy X-ray absorptiometry scan analysis.

### The Effect of Dietary Treatment on Selected Serum Biochemistries

There was no decrease in serum total protein or serum albumin concentrations after consuming dietary treatment foods for 6 months based on time or dietary treatment (**Table 2**). The concentration of serum urea nitrogen decreased (*P*<0.01) in dogs of all three treatment groups after 6 months of food consumption, but there were no differences based on dietary treatment. The change in serum creatinine concentrations across time (increased; data not shown) in dogs consuming the increased (n-3) and (n-6) FA food that was enriched in MCT, or experimental food with increased (n-3) FA content alone, was greater (*P*<0.01 and *P* = 0.03, respectively) than the change across time in dogs consuming control food (decreased). However, changes in serum creatinine concentrations were within the reference interval and biologically insignificant. Serum phosphorus concentrations increased (*P* = 0.03) in dogs of all three treatment groups after 6 months of food consumption, but there were no differences based on dietary treatment. Again, changes in serum phosphorus concentrations were within the reference interval and biologically insignificant. Serum triglyceride concentrations approximately doubled (*P*<0.01) in dogs of all three treatment groups after 6 months of food consumption, but there were no differences based on dietary treatment. Serum cholesterol concentrations also increased (*P*<0.01) in dogs of all three treatment groups after 6 months of food consumption, although the change across time (data not shown) was less in dogs consuming the increased (n-3) and (n-6) FA food that was enriched in MCT than in control dogs (*P* = 0.01) or dogs consuming experimental food with increased (n-3) FA content alone (*P* = 0.03).

### The Effect of Dietary Treatment on Concentrations of FA in Serum Determined by Gas Chromatography of FA Methyl Esters

The FA composition of serum in dogs from all three treatment groups at baseline (initial) were the same (**Table 3**). Subsequent supplementation of food with 0.6 or 1.5% fish oil produced proportionate increases in FA concentrations of EPA and DHA in dogs consuming the two experimental foods. Dogs consuming test food for 6 months with 1.5% fish oil, reduced AA, added MCT from coconut oil, and added corn oil had greater increases in serum lauric, myristic, and LA FA concentrations compared to dogs consuming control food or food enriched with 0.6% fish oil. These dogs also had decreased palmitic, palmitoleic, oleic and AA FA concentrations.

**Table 3:** Serum concentrations of selected fatty acids (mean ± SEM) in dogs at baseline (initial) and after consuming control* or treatment foods for 194 days.†

	Control Food		Increased (n-3) FA Food	Increased(n-3) and (n-6)FA Food	Two-way ANOVA Analysis(*P* values)		
**Number of Animals, N**	13		12	13	**Diet Main Effect**	**Time Main Effect**	**Diet by Time Main Effect**
**Added Fish Oil, %**	0		0.6	1.5			
**Added L-carnitine, mg/kg**	0		300	300			
**Added Coconut and Corn Oils, Reduced Animal Fat (+,–)**	–		–	+			
**Fatty Acids:**							
Lauric acid [12∶0]InitialFinalChange	0.07±0.040.20±0.07^b^0.13±0.05^b^		0.07±0.030.28±0.09^b^0.22±0.07^b^	0.10±0.050.68±0.03^a^0.57±0.07^a^	<0.01<0.01	<0.01	<0.01
Myristic Acid [14∶0]InitialFinalChange	0.63±0.030.85±0.03^b^0.24±0.05^b^		0.59±0.040.99±0.11^b^0.41±0.11^a,b^	0.73±0.161.18±0.08^a^0.44±0.19^ a^	<0.01<0.01	<0.01	
Palmitic Acid [16∶0]InitialFinalChange	30.57±0.7934.29±0.81^a^3.87±0.97 ^a^		30.86±1.2435.75±2.16^a^5.07±2.29^a^	30.77±1.8427.44±0.80^b^−3.33±2.11^b^	<0.01<0.01	<0.01	<0.01
Stearic Acid [18∶0]InitialFinalChange	63.32±1.5769.91±1.77^a,b^7.70±1.96^a,b^		65.34±2.6676.12±6.45^a^11.15±5.96^a^	64.03±3.7466.37±2.51^b^2.34±4.07^b^	0.070.07	<0.01	
**∑ SFA^£^**InitialFinalChange	94.35±3.78104.8±4.07^a,b^10.43±5.51^a,b^		97.28±4.06113.6±4.22^a^16.34±5.81^a^	95.61±4.0495.63±4.04^b^0.02±5.70^b^	<0.01<0.01	<0.01	
Palmitoleic [16∶1]InitialFinalChange	2.03±0.092.35±0.120.32±0.15^a^		2.01±0.152.39±0.160.38±0.19^a^	2.33±0.142.01±0.15−0.32±0.16^b^	<0.01		
Oleic [18∶1]InitialFinalChange	19.97±0.7123.02±0.72^a^3.13±0.99^a^		19.73±0.9122.87±1.59^a^3.14±1.63^a^	19.67±0.7817.84±0.67^b^−1.83±1.05^b^	<0.01<0.01	<0.01	<0.01
**∑ MUFA^¥^**InitialFinalChange	22.1±0.9125.4±0.96^a^3.3±1.30^a^		21.8±0.9725.3±1.01^a^3.5±1.40^a^	21.9±0.9719.7±0.97^b^−2.1±1.37^ b^	<0.01<0.01	<0.01	<0.01
LA [18∶2 (n-6)]InitialFinalChange		45.39±1.4744.59±1.25^b^−0.80±1.27^b^	45.67±2.0949.23±4.4^a,b^3.66±3.36^a,b^	45.20±1.9652.72±1.41^a^7.52±2.13^a^	<0.01<0.01	<0.01	
γLA [18∶3 (n-6)]InitialFinalChange		0.26±0.030.44±0.010.18±0.03	0.28±0.030.45±0.010.17±0.03	0.32±0.010.43±0.010.11±0.01		<0.01	
αLA [18∶3 (n-3)]InitialFinalChange		2.63±0.122.79±0.09^e^0.17±0.15	2.61±0.163.15±0.22^d^0.54±0.23	2.53±0.132.99±0.12^d,e^0.47±0.17	<0.01	<0.01	<0.01
AA [20∶4 (n-6)]InitialFinalChange		48.30±1.2655.58±1.44^a^7.28±1.57^a^	50.02±2.5551.51±4.99^a^1.49±4.35^a^	49.40±2.5938.18±1.76^b^−11.22±2.69^b^	<0.01<0.01		<0.01
EPA [20∶5 (n-3)]InitialFinalChange		1.33±0.101.68±0.10^c^0.37±0.05^c^	1.26±0.123.77±0.35^b^2.54±0.34^b^	1.25±0.096.56±0.38^a^5.31±0.35^a^	<0.01<0.01	<0.01	<0.01
DHA [22∶6 (n-3)]InitialFinalChange		1.66±0.103.51±0.23^c^1.85±0.43^c^	1.49±0.078.86±0.52^b^7.38±0.45^b^	1.47±0.1311.06±0.50^a^9.59±0.44^a^	<0.01<0.01	<0.01	
**∑ PUFA^¶^**InitialFinalChange		104.0±3.99114.5±4.2010.5±5.69	105.8±4.20122.5±4.3616.7±6.01	104.2±4.17117.1±4.1712.9±5.89		<0.01	
**PUFA:SFA ratio**InitialFinalChange		1.10±0.011.08±0.01^b^−0.02±0.07^b^	1.10±0.011.08±0.01^b^−0.02±0.09^b^	1.09±0.011.22±0.01^a^0.13±0.13^a^	<0.01<0.01	<0.01	<0.01
**∑ (n-6)FA^#^**InitialFinalChange		98.4±3.59106.6±3.87^a^8.14±5.24^a^	100.5±3.87106.8±4.02^a^6.29±5.54^a^	98.8±3.8496.4±3.84^b^−2.43±5.42^b^	<0.01<0.01	<0.01	
**∑(n-3)FA^‡^**InitialFinalChange		5.56±0.507.91±0.54^c^2.35±0.73^c^	5.32±0.5415.8±0.56^b^10.44±0.77^b^	5.36±0.5420.73±0.54^a^15.37±0.76^a^	<0.01<0.01	<0.01	<0.01
**(n-6):(n-3) FA ratio**InitialFinalChange		18.18±0.3313.62±0.35^a^−4.56±0.48^a^	19.07±0.356.46±0.37^ b^−12.6±0.51^b^	18.53±0.354.69±0.35^c^−13.84±0.50^c^	<0.01<0.01	<0.01	<0.01

a,b,c Means with different superscripts within a row are different at *P*≤0.05.

d,e Means with different superscripts within a row are different at *P* = 0.06.

† Fatty acid concentrations in serum, determined by gas chromatography of FA methyl esters, are expressed as mg/dL.

* ^,£, ¥, ¶,#,‡^ See Table 1 for rest of key.

Serum FA were grouped according to their degree of saturation (sum of SFA, MUFA, and PUFA), and class of FA [sum of (n-3) and (n-6) FA] (**Table 3**). Although the sum of serum (n-3) FA increased with fish oil supplementation, the sum of serum PUFA did not change based on dietary treatment. The sums of serum SFA and PUFA both increased in dogs consuming control and increased (n-3) FA foods; thus, the PUFA:SFA ratio was unchanged. In dogs consuming increased (n-3) and (n-6) FA food that was enriched in MCT, the increase in sum of serum PUFA was not offset by a change in sum of serum SFA so the PUFA:SFA ratio increased (*P<0.01*).

### The Effect of Dietary Treatment on Serum Metabolomic Profiles

Carnitine and its metabolites that were significantly affected within and among dogs maintained on the three test foods are presented in **Table 4** **.** Serum carnitine levels were significantly increased by dietary supplementation with 300 mg/kg as fed. In addition, multiple carnitine metabolites (acetyl-, stearoyl-, propionyl-, succinyl-, glutaroyl-, 2-methylbutyroyl-, and isovaleryl-carnitine) increased with time (*P<0.05)* relative to baseline concentrations in dogs receiving carnitine-supplemented diets. However, the long-chain acylcarnitine (stearoyl-carnitine) was significantly (*P<0.05)* increased over baseline only in dogs receiving fish oil at the 0.6% supplementation rate with no added MCT. Deoxycarnitine concentrations decreased across time in all dog treatment groups (*P<0.05).* One carnitine metabolite (2-methylbutyroylcarnitine) was positively correlated to creatinine concentration (*P<0.01*).

**Table 4:** Carnitine metabolites of dogs after consuming control* or treatment foods for 194 days.†

	Control Food	Increased (n-3) FA Food	Increased(n-3) and (n-6)FA Food	Two-way ANOVA Analysis(*P* values)		
**Number of Animals, N**	13	12	13	**Diet Main Effect**	**Time Main Effect**	**Diet by Time** **Main Effect**
**Added Fish Oil, %**	0	0.6	1.5			
**Added L-carnitine, mg/kg**	0	300	300			
**Added Coconut and Corn Oils, Reduced** **Animal Fat (+,–)**	–	–	+			
Carnitine	0.71^a^	1.22^b^	1.12	<0.10		<0.05
Deoxy-carnitine	0.95	0.95	0.89		<0.05	
Acetyl-carnitine	1.08	1.57^a^	1.48^a^		<0.05	<0.05
Stearoyl-carnitine	1.49	4.00^a^	0.84		<0.05	<0.05
Propionyl-carnitine	1.22	1.58^a^	1.85^a^		<0.05	<0.05
Succinyl-carnitine	1.04	1.25^a^	1.39^a^		<0.05	<0.10
Glutaroyl-carnitine	1.36^a^	1.62^a^	1.63^a^		<0.05	
2-methylbutyroyl-carnitine	1.10	2.03^a^	1.87^a^		<0.05	<0.05
Isovaleryl-carnitine	0.99	1.71^a^	1.84^a^	<0.05	<0.05	<0.05
Hydroxyisovaleroyl-carnitine	0.90^b^	1.01	1.02			

* PrescriptionDiet® k/d®, Hill’s Pet Nutrition, Inc.

† Using serum values from baseline, each animal served as its own control. Data are presented relative to baseline as fold change. All data were log-transformed prior to statistical analysis.

a Indicates significantly increased or decreased fold-change between the diet group shown and baseline at *P*<0.05.

b Indicates fold-change values that are trends but missed the significant cutoff (*P*>0.05, *P*<0.10).

Selected lysophospholipids from serum metabolomic profiles that showed statistically significant differences among dogs maintained on the three test foods are presented in **Table 5** **.**

**Table 5:** Lysophospholipids of dogs after consuming control* or treatment foods for 194 days.†

	Control Food	Increased (n-3) FA Food	Increased(n-3) and (n-6)FA Food	Two-way ANOVA Analysis(*P* values)		
**Number of Animals, N**	13	12	13	**Diet Main Effect**	**Time Main Effect**	**Diet by Time** **Main Effect**
**Added Fish Oil, %**	0	0.6	1.5			
**Added L-carnitine, mg/kg**	0	300	300			
**Added Coconut and Corn Oils, Reduced** **Animal Fat (+,–)**	–	–	+			
1-linoleoyl-glycerophosphoethanolamine	0.81	0.72^a^	0.98		<0.05	
2-linoleoyl-glycerophosphoethanolamine	0.87^a^	0.85^b^	0.97	<0.05	<0.05	
1-arachidonoyl-glycerophosphoethanolamine	0.96	0.78^b^	0.65^a^	<0.05	<0.05	
2-arachidonoyl-glycerophosphoethanolamine	0.85	0.76^b^	0.92	<0.05	<0.10	
1-arachidonoyl-glycerophosphocholine	1.75	0.96	1.06			
2-arachidonoyl-glycerophosphocholine	1.80	2.05	0.81			
1-docosahexaenoyl-glycerophosphocholine	1.73^b^	3.77^a^	2.79^a^	<0.05	<0.05	

* PrescriptionDiet® k/d®, Hill’s Pet Nutrition, Inc.

† Using serum values from baseline, each animal served as its own control. Data are presented relative to baseline as fold change. All data were log-transformed prior to statistical analysis.

a Indicates significantly increased or decreased fold-change between the diet group shown and baseline at *P*<0.05.

b Indicates fold-change values that are trends but missed the significant cutoff (*P*>0.05, *P*<0.10).

Supplementation of food with fish oil affected concentrations of several lysophopholipids. In particular, the glycerophosphoethanolamines (GPE) 1-arachidonoyl- and 2-arachdidonoyl-GPE, but also 1-linoleoyl- and 2-linoleoyl-GPE, showed significant dietary treatment and time main effects. Of particular note, the two dog groups receiving fish-oil supplementation showed decreased concentrations across time. In contrast to the GPE, the glycerophosphocholine (GPC) containing DHA (1-docosahexaenoyl-GPC) showed both dietary treatment and time main effects. Its concentration was dramatically increased across time although it was significantly greater in dogs receiving 0.6% vs. 1.5% fish oil supplementation rate in a reverse dose-response manner. There was no effect of dietary treatment or time on glycerophosphoinositol concentrations (data not shown).

### The Effect of Age on Circulating Fatty Acid and Carnitine Metabolite Concentrations

At baseline, the ratio for geriatric to mature adults dogs of PUFA to SFA ratios was significantly decreased (*P*<0.05; **Table 6**) because the ratio of PUFA to SFA in mature dogs was greater than for geriatric dogs. This was most likely because the concentrations of SFA were greater in geriatric dogs (not significant at *P*<0.05*)*. No other significant differences were noted at baseline for relative ratios of serum concentrations of FA in geriatric vs. mature dogs. There was no age by diet interaction; hence FA concentrations for mature or geriatric dogs consuming the three test foods were combined across time. At the end of the study, the increases associated with supplemented food were much greater than the increases associated with younger age. None-the-less, increasing age was significantly associated with decreasing serum EPA (*P*<0.05), DHA (*P*<0.10), and sum of the (n-3) FA (*P*<0.10) concentrations (**Table 6**).

**Table 6:** Relative ratios of serum concentrations of selected fatty acids and carnitine metabolites in geriatric dogs (>7 yr) vs. mature adult dogs (≤7 yr) at baseline (initial) and after consuming control* or treatment foods for 194 days.

	Relative Concentration Ratios† for Geriatric to Mature Adult Dogs		
	Initial(Baseline)	Final(After 194 Days)	Combined§
**Fatty Acids:**			
∑ SFA^£^	1.01	0.96	0.98
∑ MUFA^¥^	0.92	0.99	0.96
LA [18∶2 (n-6)]	0.98	1.04	1.01
γLA [18∶3 (n-6)]	1.02	0.97	0.99
αLA [18∶3 (n-3)]	0.97	1.00	0.99
AA [20∶4 (n-6)]	0.96	0.91	0.94
EPA [20∶5 (n-3)]	0.90	0.94	0.93^a^
DHA [22∶6 (n-3)]	1.13^b^	0.91^b^	0.94^b^
∑ PUFA^¶^	0.98	0.98	0.98
PUFA:SFA ratio	0.97^a^	1.02^a^	1.00
∑ (n-6)FA^#^	0.98	0.98	0.98
∑(n-3)FA^‡^	0.99	0.94	0.95^b^
(n-6):(n-3) FA ratio	0.99	1.08	1.01
**Carnitine Metabolites:**			
Carnitine	0.95	1.09	1.02
Deoxy-carnitine	0.90^b^	0.95	0.92^b^
Acetyl-carnitine	0.96^b^	0.94	0.95
Stearoyl-carnitine	0.94	1.31	1.17
Propionyl-carnitine	0.89	1.00	0.96^b^
Succinyl-carnitine	0.81^a^	0.87^a^	0.84^a^
Glutaroyl-carnitine	0.95	0.85^b^	0.89 b
2-methylbutyroyl-carnitine	0.78^a^	0.95	0.88^a^
Isovaleryl-carnitine	1.12	1.25	1.20
Hydroxyisovaleroyl-carnitine	0.76	0.78	0.77^a^

† The ratios are the average concentration of an analyte for dogs in the geriatric group divided by average concentration of the same analyte for dogs in the mature adult group.

§ Because there was no age by diet interaction, concentrations for dogs consuming different diets are combined across time.

a There was a significant effect of age on these ratios (*P*≤0.05).

b There was an effect of age on these ratios (*P*>0.05, *P*<0.10).

* ^,£, ¥, ¶,#,‡^ See Table 1 for rest of key.

At baseline, the relative concentration ratios for geriatric to mature adult dogs were less than one for carnitine and most carnitine metabolite concentrations. Succinyl-carnitine and 2-methylbutyroyl-carnitine were significantly decreased in dogs over 7 years of age (*P*<0.05; **Table 6** **)** as were deoxy-carnitine and acetyl-carnitine at *P*<0.10**.** There was no age by diet interaction; hence serum carnitine and its metabolite concentrations for mature or geriatric dogs consuming the three test foods were combined across time. Increasing age was significantly (*P*<0.05) associated with decreasing serum succinyl-carnitine, 2-methylbutyroyl-carnitine, and hydroxyisovaleroyl-carnitine concentrations, whereas deoxy-carnitine, propionyl-carnitine, and glutaroyl-carnitine concentrations were decreased at P<0.10 (**Table 6** **).** There was no statistically significant increase in any carnitine metabolite concentration as a result of age.

Adding dietary MCT, fish oil, and L-carnitine attenuated the normal effects of aging on circulating concentrations of these supplemented FA (**Table 3**) in that FA concentrations were all increased as a result of supplementation. The PUFA to SFA ratio was greater as a result of supplementation in dogs consuming the increased (n-3) and (n-6) FA food that was enriched in MCT, mainly because the SFA concentrations did not increase as they did in dogs consuming control food or experimental food with increased (n-3) FA content alone. Adding L-carnitine to the food counterbalanced the effects of aging on circulating concentrations of most carnitine metabolites in that fold change relative to baseline was significantly greater than one (*P*<0.05; **Table 4** **).**

## Discussion

### All Foods Maintained Total-lean-body Weight and Biomarkers of Protein Adequacy

Canine Prescription Diet® k/d® was used as the control food and base formula for treatment foods in this study. It has reduced protein (13.5% as fed), phosphorus, and sodium concentrations compared to other canine maintenance foods, and contains greater levels of non-protein calories (18.0% fat; 56.9% carbohydrate). For comparison, Science Diet® Canine Adult® has 23.0% protein, 14.2% fat, and 49.0% carbohydrate. Animal feeding tests using AAFCO [22] procedures substantiate that Canine k/d® provides complete and balanced nutrition for maintenance of adult dogs. In the current study, all dogs maintained total- and lean-body weight, serum total protein and serum albumin concentrations. All values were within normal ranges for adult dogs [25]. These results support the findings of a previous study [26] that this diet provides adequate protein nutrition for maintenance of adults dogs.

### Adding Specific FA to the Diet Changed those FA in Circulation

The ratio of PUFA to SFA was significantly greater in mature dogs compared with geriatric dogs at baseline, likely because SFA concentrations were greater in geriatric dogs. The pre-trial food was relatively low in fat, including (n-3) FA, compared to test foods such that other FA differences between age groups at baseline were not detectable. Adding 1.5% fish oil plus MCT to the food significantly increased the ratio of PUFA to SFA in all dogs, with the net effect that all dogs looked more like younger dogs. Younger dogs were more efficient at increasing (n-3) FA concentrations than older dogs, as evidenced by the relative ratios of serum (n-3) FA concentrations in geriatric vs. mature adult dogs being less than one at the end of the study. Adding 0.6 or 1.5% fish oil produced proportionate increases in serum FA concentrations of EPA and DHA in dogs consuming the two treatment foods. Fatty acid changes determined by metabolomic analysis of serum confirmed FA changes determined by gas chromatography of FA methyl esters (data not shown). These results reflect the effect of dietary dose rather than ratio of (n-6) to (n-3) FA on serum FA concentrations, and are consistent with what we found in previous studies [9], [12]. In previous studies the addition of 2% fish oil to the diet reduced the relative abundance of serum AA and LA in dogs. In the current study, the addition of 0.6% dietary fish oil increased serum EPA and DHA concentrations, but was not associated with a concomitant decrease in serum AA or LA. Adding 1.5% fish oil plus coconut and corn oils to the food decreased serum AA in dogs, but increased LA concentrations compared to those of dogs eating control food.

Adding 1.5% fish oil plus MCT to the food caused small albeit significant increases in serum lauric [12∶0] and myristic [14∶0] FA, but much larger decreases in palmitic [16∶0], palmitoleic [16∶1] and oleic [18∶1] FA concentrations. Stearic acid [18∶0] concentrations increased in dogs of all treatment groups, with significantly greater concentrations in dogs consuming treatment food supplemented with 0.6% fish oil.

It is difficult to determine from our results whether MCT were hydrolyzed more quickly, sparing the (n-3) FA from entering oxidative pathways. Highly unsaturated FA are favored for esterification into phospholipids, which makes them more abundant in phosphoglycerides than in triacylglycerols and makes analysis of phospholipids a preferred approach for evaluating highly unsaturated FA contents [27]. In our study, however, we did not fractionate plasma lipids into subclasses prior to analysis.

In our study, the ratio of MCT to fish oil was approximately 1.33. In the Simoens et al. [18] study of parenteral nutrition, lipid emulsions were 50% MCT, 40% long-chain triacylglycerol, and 10% fish oil (wt:wt:wt). In the Carpentier et al. [20] study, the emulsions were 80% MCT and 20% fish oil. The content of (n-3) PUFA was assessed in plasma, leukocyte and platelet phospholipids in both of these human studies, and infusion of MCT:fish oil emulsions allowed rapid enrichment of cells with (n-3) PUFAs.

Studzinski et al. [28] reported that greater rates of FA oxidation were achieved in aged dogs by exogenously adding MCT to their normal diet. A structured triglyceride containing 95% caprylic acid [C8∶0] and 5% capric acid [C10∶0] was added to the food and fed at 2 g/kg body weight in their study. In a follow up study, Taha et al. [21] showed that dietary enrichment with MCT increased DHA concentrations in brain total lipids, phospholipids, and unesterified FA in aged dogs. Because the MCT supplement did not contain PUFA, the increase in DHA was likely due to tissue redistribution from adipose to brain. Although we did not measure FA oxidation or tissue incorporation of FA in our study, increased DHA concentrations in dogs fed 1.5% fish oil and MCT may reflect increased dietary DHA as well as spared oxidation of DHA by dietary MCT. Future studies are needed to control for the amount of added fish oil in the presence of MCT.

### Adding Dietary Carnitine Increased Serum Carnitine and its Metabolites

At baseline, several of the serum carnitine metabolite concentrations were significantly decreased in geriatric dogs. We found that supplementing with L-carnitine resulted in increased concentrations of circulating carnitine metabolites to counterbalance the effects of aging. Younger dogs were more efficient at increasing carnitine metabolite concentrations than older dogs, as evidenced by the number of statistically significant relative concentration ratios that were less than one at the end of the study.

The acylation state of carnitine in serum reflects the composition of the cytosolic acylcarnitine pool, and thus, the equilibrium between acyl-CoA and acylcarnitine species. Future studies in dogs are needed to determine if maintaining circulating concentrations of carnitine metabolites long term confers age-related health benefits. For example, a recent study assessing the impact of acylcarnitine acyl chain length showed that longer acyl chain length significantly predicts poorer physical function and worsened anemia in dialysis patients [29].

### Serum Docosahexaenoyl-glycerophosphocholine (DHA-GPC) Concentration was Increased by Feeding Fish Oil

Phospholipids are major constituents of biological membranes. Membrane lipids are constantly synthesized and degraded (metabolic turnover) via a collection of specific hydrolases stored in lysosomes, including phospholipase A_1_ and A_2_. These hydrolases remove FA from membrane glycerophospholipids; these FA are then used in the synthesis of eicosanoids. Eicosanoids derived from EPA are considered less inflammatory than those derived from AA [14]. The remnant molecule, a glycerophospholipid with one FA removed, is called a lysophospholipid; these metabolites are biologically important as well.

Previously, we reported that serum concentrations of DHA-GPC were significantly increased in a dose-response manner in Beagle dogs following dietary fish oil enrichment [13]. Those lysophospholipid results were in agreement with the notion that DHA is incorporated into inflammatory cell phospholipids, partly at the expense of AA. A clinical trial in humans also showed that plasma EPA- and DHA-lysophosphatidylcholine concentrations were significantly increased with EPA/DHA supplementation [30]. In the current study, serum DHA-GPC was increased by feeding fish oil, but the addition of MCT attenuated the dose-response relationship. The mechanism of these relationships remains unknown.

In the immune system, lysophospholipid signaling may have anti-inflammatory roles through modulating cytokines, lipid mediators, and transcription factors in epithelial cells [31]. Thus, DHA-GPC may express anti-inflammatory activity through multiple actions, including suppression of nitric oxide, TNF-α inhibition of 5-lipoxygenase activity, as well as via formation of maresin or protectin D via a series of metabolic activation pathways. Of major importance, Huang et al. [32] showed that the diverse properties of the GPC class of lysophospholipids are highly dependent on the type of FA attached to the GPC. Hung et al. [33] suggested that 2-DHA-GPC may be more efficient than 1-DHA-GPC as an anti-inflammatory agent when administered orally to mice. DHA migrates very easily from the *sn*-2 position of GPC, which could be considered as the physiological form of DHA-GPC, to the *sn*-1 position, which is much more stable [34].

Kono et al. [35] have shown that enteral diets enriched with both (n-3) FA and MCT are superior to diets enriched with (n-3) FA alone for the therapy of chemically induced colitis in rats. Together they inhibit the expression of inflammatory cytokines/chemokines in the colonic tissue, the production of those mediators by activated macrophages, and the accumulation of activated neutrophils in the colon, all of which ameliorated colonic injury.

### Creatinine Concentrations were Increased in Older Dogs and in Dogs Consuming Treatment Foods, but Remained within the Normal Reference Interval

In the absence of dehydration, circulating creatinine concentration is often used as an indicator of glomerular filtration rate, and by extrapolation, a marker to assess renal function. There was a significant positive correlation between circulating creatinine concentration and total-lean-body weight. When this relationship was used as a covariate, circulating creatinine concentrations were increased in older dogs. We also showed a small but significant increase in circulating creatinine concentration in dogs consuming treatment foods. Although there was a significant correlation between 2-methylbutyroyl carnitine and creatinine, we found no effect of changing circulating concentrations of carnitine or its metabolites on creatinine concentrations.

In conclusion, supplementation of dogs with L-carnitine, MCT, and EPA and DHA FA counterbalanced changes in these serum biochemistries as dogs aged, although younger dogs were more efficient in their accrual. Importantly, changes in circulating metabolites were not associated with any detectable change in renal function in healthy dogs with normal creatinine concentrations.
